# Explainable AI-driven depression detection from social media using natural language processing and black box machine learning models

**DOI:** 10.3389/frai.2025.1627078

**Published:** 2025-09-11

**Authors:** Sidra Hameed, Muhammad Nauman, Nadeem Akhtar, Muhammad A. B. Fayyaz, Raheel Nawaz

**Affiliations:** ^1^Faculty of Computing, The Islamia University of Bahawalpur, Punjab, Pakistan; ^2^Department of Information Technology, FCIT, University of the Punjab, Lahore, Pakistan; ^3^OTEHM, Manchester Metropolitan University, Manchester, United Kingdom; ^4^Pro VC-Staffordshire University, Staffordshire, United Kingdom

**Keywords:** mental illness detection, natural language processing, machine learning, explainable artificial intelligence, Local Interpretable Model-Agnostic Explanations (LIME)

## Abstract

**Introduction:**

Mental disorders are highly prevalent in modern society, leading to substantial personal and societal burdens. Among these, depression is one of the most common, often exacerbated by socioeconomic, clinical, and individual risk factors. With the rise of social media, user-generated content offers valuable opportunities for the early detection of mental disorders through computational approaches.

**Methods:**

This study explores the early detection of depression using black-box machine learning (ML) models, including Support Vector Machines (SVM), Random Forests (RF), Extreme Gradient Boosting (XGB), and Artificial Neural Networks (ANN). Advanced Natural Language Processing (NLP) techniques TF-IDF, Latent Dirichlet Allocation (LDA), N-grams, Bag of Words (BoW), and GloVe embeddings were employed to extract linguistic and semantic features. To address the interpretability limitations of black-box models, Explainable AI (XAI) methods were integrated, specifically the Local Interpretable Model-Agnostic Explanations (LIME).

**Results:**

Experimental findings demonstrate that SVM achieved the highest accuracy in detecting depression from social media data, outperforming RF and other models. The application of LIME enabled granular insights into model predictions, highlighting linguistic markers strongly aligned with established psychological research.

**Discussion:**

Unlike most prior studies that focus primarily on classification accuracy, this work emphasizes both predictive performance and interpretability. The integration of LIME not only enhanced transparency and interpretability but also improved the potential clinical trustworthiness of ML-based depression detection models.

## 1 Introduction

Mental disorders, also known as psychiatric disorders, encompass a wide range of conditions that disrupt thoughts, emotions, and behaviors, often impairing an individual's ability to function in daily life (Orabi et al., 2018; Zhang T. et al., 2022). These include depression, anxiety, schizophrenia, and bipolar disorder, with causes rooted in genetic, environmental, and biological factors. Among these, depression is particularly prevalent and debilitating, affecting health, relationships, and productivity. Despite the availability of treatments like psychotherapy and pharmacotherapy (Ive et al., 2020), depression remains a global public health concern. According to the World Health Organization, over 322 million people suffer from depression worldwide, yet many cases go undiagnosed due to stigma and limited access to mental health services, especially in low- and middle-income countries (Velupillai et al., 2018; World Health Organization, 2021).

To address these challenges, researchers are increasingly leveraging digital data sources—particularly social media—to detect mental health issues using Natural Language Processing (NLP) and Machine Learning (ML) techniques (World Health Organization, Regional Office for the Eastern Mediterranean). Social media platforms like Twitter (X), Reddit, and Facebook offer real-time insights into users' emotions, behavior, and potential mental distress. Recent studies have demonstrated the potential of analyzing linguistic and behavioral cues to predict mood disorders and related symptoms, such as stress, self-harm, and emotional deterioration, without requiring traditional clinical assessments (Yazdavar et al., 2020; Olusegun et al., 2023; AbuRaed et al., 2024; Chancellor and De Choudhury, 2020). These digital approaches offer scalable, non-intrusive alternatives to traditional mental health diagnostics, especially in underserved populations.

Natural Language Processing (NLP) is a subfield of Artificial Intelligence (AI) that has facilitated various tasks in recent years, including the management and analysis of large amounts of textual data, information extraction, sentiment analysis, emotion detection, and mental health surveillance, among others (Steinkamp and Cook, 2021a). Feature extraction techniques in NLP are essential for transforming unstructured textual data into structured numerical representations, thereby enabling machine learning models to perform tasks such as classification, sentiment analysis, and topic modeling. Traditional methods such as Bag of Words (BoW), Term Frequency-Inverse Document Frequency (TF-IDF), and N-grams convert text into sparse feature vectors by capturing lexical patterns and word co-occurrence frequencies (Salton and Buckley, 1988; Jurafsky and Martin, 2000). While effective for basic NLP tasks, these approaches cannot capture deeper semantic relationships and contextual nuances. To address these limitations, more advanced feature extraction techniques such as Word2Vec, GloVe (Pennington et al., 2014), and contextual embeddings from transformer-based models like BERT (Devlin et al., 2019) have been developed. These methods represent words in dense vector spaces and incorporate semantic and syntactic context, significantly improving performance across a wide range of downstream NLP applications.

In general, users can convey their emotions through various written formats, such as posts on social media platforms, interview transcripts, and professional notes that include patient descriptions of their mental states. X (formerly known as Twitter) is most commonly known as a platform for micro-blogging because it has a straightforward user interface that enables the publication of short stories of no more than 280 characters. Tweets posted by virtually every user are available to the general public and can be retrieved using the user's own X API (Govindasamy and Palanichamy, 2021). X enables users to analyse and understand current events and trends, regardless of their geographical location. More recently, the research work focused on determining whether a person is depressed by analyzing their tweets. More precisely, Sentiment analysis can determine whether a piece of writing has been produced in a positive, negative, or neutral tone. Comments and posts from other X users can reveal whether a user is happy or sad at any given moment. Each tweet is evaluated based on its positive, negative, or neutral sentiments. The NLP system may classify tweets as either depressive or non-depressive by identifying depressive symptoms.

More recently, black box ML algorithms have demonstrated exceptional performance in text classification and analysis, yielding accurate and efficient results across various applications. However, their intrinsic black box nature poses notable challenges to transparency, interpretability, and trustworthiness, especially in critical domains where comprehensible decision-making processes are essential (Cesarini et al., 2024; Jahromi et al., 2024). The lack of transparency in ML and its black box nature are significant issues in its implementation in critical domains, such as healthcare (Khan et al., 2024; Weerts et al., 2019; Chakraborty et al., 2021; Kawakura et al., 2022; Nauman et al., 2021). Explainable Artificial Intelligence (XAI) is a subdomain of AI that aims to improve transparency by explaining the internal decision-making processes of such models (Chen et al., 2021).

One the contrary, a few known efforts to explain the black box models in literature include SHAP (Chelgani et al., 2023) and LIME (Hakkoum et al., 2020). Recent research in explainability focuses on revealing the primary features that significantly impact a model's decision-making process (Zhang Y. et al., 2022). As AI-based systems only make predictions without explaining their rationale, there is a need for mechanisms to explain and interpret their decisions. Furthermore, Local Interpretable Model-agnostic Explanations (LIME) is an explainability technique used to interpret the predictions made by machine learning models. The LIME technque approximates a complex model with a local, interpretable one around the prediction to be explained, thereby offering insights into the model's behavior on individual predictions (Ribeiro et al., 2016b). This method is particularly valuable in domains where understanding the decision-making process is crucial, such as healthcare and mental health diagnosis, as it helps build trust and provides transparency in the model's decisions (Ribeiro et al., 2016a). By applying LIME to the ML models, we can identify which features contribute most to the predictions, thus making the model's decisions more understandable and actionable for stakeholders.

Recent research on depression detection from social media platforms like Twitter and Reddit has shown that ML and deep learning models—such as CNNs, LSTMs, and BERT—are effective in identifying mental health indicators from user-generated content (Amanat et al., 2022b; Ji et al., 2022b). However, key gaps remain: many models operate as black boxes with limited interpretability, which is problematic in clinical contexts requiring transparency (Guo et al., 2023a; Ibrahimov and Ali, 2024). Most studies rely on a single feature representation method, overlooking the benefits of combining traditional and semantic features. Comparative evaluations across diverse models ranging from classical ML to deep neural networks are rare, limiting insight into their relative performance. Additionally, while XAI tools like LIME and SHAP are gaining traction, their integration into end-to-end depression detection systems remains limited. Finally, many datasets are weakly labeled, often based on heuristics or self-reports without clinical validation, undermining the reliability of resulting models (Chancellor and De Choudhury, 2020; Yazdavar et al., 2020).

This research distinguishes itself from existing literature by employing multiple feature extraction techniques and the LIME (Ribeiro et al., 2016b) method to elucidate the internal decision-making processes of machine learning models. This work focuses on interpreting the detection decisions made by ML models to enhance early mental disorder detection and support healthcare professionals. The research results will enable physicians to identify life-threatening diseases in their early stages, ultimately facilitating a healthier society. This work aims to leverage advanced ML techniques and XAI to facilitate the early detection of myocardial infarction through the analysis of X data. By addressing the gap in understanding how social media conversations can be leveraged for mental health insights, this research contributes novel methodologies for pre-processing data, feature extraction, and model interpretation.

The main contributions of this research work are as follows:

- We applied LIME explainability uniformly to 28 different feature-classifier combinations (7 feature extraction methods × 4 classifiers), rather than limiting interpretation to the single highest-accuracy model as in most prior studies. This research work provides a comprehensive view of how different models make predictions in the depression detection context.
- The research findings are structured to separately rank feature extraction methods and classifiers, eliminating cross-category confusion and allowing researchers to see the independent effect of each.
- Beyond standard tokenisation and normalization, the proposed pipeline includes slang expansion, emoticon-to-text conversion, and a mental health-specific stopword list, in particular, tailored to the noisy and abbreviated nature of depression-related social media posts.
- The research connected linguistic patterns highlighted by LIME to known depression-related cues in psychology and linguistics literature, providing actionable insights for mental health professionals and validating black box ML model outputs beyond raw accuracy.
- We systematically applied LIME across all model configurations, selecting it for its model-agnostic nature, suitability for short-text explanations, and computational efficiency.

## 2 Background

### 2.1 Mental disorder detection

Modern society is plagued by a high prevalence of mental disorders, a significant source of personal and societal suffering. It is a complex, multifactorial disease influenced by several socioeconomic and clinical factors, as well as individual risk factors (Thornicroft et al., 2022). Depression is a typical mental condition that can affect functioning and cause suicidal thoughts or attempts (Jain et al., 2022). Millions of User worldwide suffer from depression each year, which is recognized as a medical condition. Persistent unhappiness or even minor stressful life events can lead to depression, illustrating the intricate relationship between mental health, NLP, ML, and AI.

Various computing algorithms for the automatic analysis and representation of human language are referred to as NLP (Cambria and White, 2014). Within Artificial Intelligence and Computer Science, the study of NLP is of utmost significance. Research into NLP employs a wide range of theoretical frameworks and methodological approaches to enable human-computer communication using natural language. NLP is an interdisciplinary field combining elements of computer science, linguistics, and mathematics, with the fundamental objective of converting human language into executable computer instructions. Natural Language Understanding and Natural Language Generation are the two basic areas of investigation in the field of NLP (Kang et al., 2020).

### 2.2 Mental disorder detection and social media

Social media's extensive use may present opportunities to lower the prevalence of undetected mental disorders. An increasing number of research projects are investigating the connection between social media and mental health. These studies attempt to determine whether there is a causal link between social media use and negative behaviors such as stress, anxiety, depression, and suicidality (Guntuku et al., 2017).

Social media networks such as X, LinkedIn, Instagram, Snapchat, and Facebook have surged in popularity, making them one of the most important sources of readily available and easily accessible information on all facets of life (Islam et al., 2018). Users of these platforms can express themselves freely, share their thoughts and feelings, and discuss any topic. Users suffering from mental illnesses, such as depression, may isolate themselves and avoid social engagement (Hemmatirad et al., 2020). However, online platforms allow users to convey their thoughts, opinions, and sentiments regarding various topics through applications such as Facebook, X, and Instagram (Islam et al., 2018).

### 2.3 Feature extraction methods

#### 2.3.1 Latent Dirichlet Allocation

Both NLP and ML make use of a probabilistic model known as Latent Dirichlet Allocation (LDA) for topic modeling. LDA is based on the assumption that each text contained within a corpus can be modeled as a mixture of a limited number of underlying themes and that each word contained within a document is taken from one of those subjects. This assumption guides LDA's operation. By utilizing the well-known LDA approach, the limit focuses on three of the seventeen Sustainable Development Goals, while simultaneously summarizing and presenting linked subtopics (Al Qudah et al., 2022).

To construct and effectively employ an LDA model, one must first ascertain the composition of the target document's latent themes, such as θ and *z*. The following is the revised Equation 1, where γ and ϕ are the parameters of the posterior distribution of θ and *z*, respectively.


(1) $$\begin{array}{l} \varphi_{ni}\infty \beta_{iw_{n}}\exp\left\{\text{Ψ}\left(\gamma_{i}\right)\right\},\gamma_{i}=\alpha_{i}+\overset{N}{\underset{n=1}{\sum }}\varphi_{ni} \end{array}$$

The LDA model's parameters were able to be estimated after the distribution of the hidden variables had been discovered, which made the process much simpler. *M* represents the total number of documents, *d* stands for the document ID, and *dni* represents the greatest possible value for *n* that can be derived from the expectation step. Equation 2 displays these three variables in their respective spots.


(2) $$\begin{array}{l} \alpha =\alpha -H{\left(\alpha \right)}^{-1}g\left(\alpha \right),\beta_{ij}\infty \overset{M}{\underset{d=1}{\sum }}\overset{N}{\underset{n=1}{\sum }}\varphi {\left(d\right)}_{ni}^{*}w{\left(d\right)}_{n}^{j} \end{array}$$

In addition, the results of making inferences about hidden variables could be utilized in calculating the target document's generation probability value, denoted by *p*_*LDA*_(*x*|β). According to LDA, a document cannot be comprehensive unless it draws from various themes because it requires drawing from a pool of ideas. In this study, these subjects were tagged and connected into thematic groups that helped distinguish between users of diabetic mobile apps who had good and negative sentiments toward them (Ossai and Wickramasinghe, 2023).

#### 2.3.2 Term frequency-inverse document frequency

It is possible to quantify the significance or relevance of string representations (words, phrases, lemmas, and so on) by making use of the TF-IDF measure, which is utilized in the disciplines of Information Retrieval and ML that are included in a collection of documents. This can be done by comparing the document to another collection of documents. A k-best selection method and a modified version of the TF-IDF-based approach are developed as part of the feature vectorization process. Text vectorization based on modified TF-IDF, pre-trained embedding based on Google News Corpus, and a deep neural network are all components of this system (Dey and Das, 2023).

The Term Frequency (TF) of a term or word indicates the proportion of the document's total words that are comprised of instances of that term, as defined in Equation 3.


(3) $$\begin{array}{l} TF=\frac{\text{\# of times term appears in doc}}{\text{\# of terms in doc}} \end{array}$$

A term's Inverse Document Frequency (IDF) reveals how frequently it appears in the total number of documents in the corpus. Equation 4 defines the equation for calculating the IDF. Words that do not appear in many papers (such as terms used in technical jargon, for example) are given more consideration than those used repeatedly throughout the entire work.


(4) $$\begin{array}{l} IDF=\log\left(\frac{\text{\# of doc in corpus}}{\text{\# of doc containing term in corpus}}\right) \end{array}$$

Multiplying a term's TF and IDF scores yields its TF-IDF, defined in Equation 5.


(5) $$\begin{array}{l} TF-IDF=TF\times IDF \end{array}$$

TF-IDF benefits many tasks involving natural language processing. For instance, search engines use it to determine how relevant a document is to a user's query. Text summarization, topic modeling, and categorization are some other applications of TF-IDF.

It's important to remember that there are several ways to determine an individual's IDF score. The logarithm to the base 10 is frequently used. However, a natural logarithm is used by some bookstores. To further prevent division by zero, a single can be added to the denominator in the following manner in Equation 6.


(6) $$\begin{array}{l} IDF=\log\left(\frac{\text{\# of doc in corpus}}{\text{\# of doc contain term in corpus}+1}\right) \end{array}$$

The TF-IDF technique is a widely utilized algorithm in the domain of text classification. The algorithm's formula is composed of two components: TF and IDF. The TF quantifies the occurrence of words within a specific class, effectively capturing their frequency in the text. In contrast, the IDF assesses the importance of a word by considering its rarity across a collection of documents, thus mitigating the influence of commonly occurring words that provide less informational value. In this study, the TF-IDF method capitalizes on the relationship between feature words and the number of texts in which appear. However, it does not account for the variation in feature word distribution across different categories, which can adversely affect classification accuracy. Despite this limitation, the TF-IDF algorithm remains a cornerstone in text classification due to its simplicity and effectiveness in various applications (Xiang, 2022).

#### 2.3.3 N-grams

N-grams are contiguous sequences of *n* items extracted from a given sample of text or speech. These sequences are fundamental in various NLP applications, such as text prediction, language modeling, and information retrieval. The strength of N-grams lies in their ability to model local context within text sequences effectively, capturing the dependencies between words or characters, which is crucial for tasks like machine translation and speech recognition (Manning and Schütze, 1999).

#### 2.3.4 Bag of words

Bag of Words (BoW) model is a fundamental method in NLP and IR, representing text data as a collection of words without considering grammar or word order (Harris, 1954). The BoW model involves creating a vocabulary from all unique words in a corpus and then representing each document as a vector based on the frequency of each word within the document. This simple yet powerful technique has been widely used in tasks such as document classification, sentiment analysis, and IR, as it effectively captures the presence of words in documents, which can be indicative of their content (Manning and Schütze, 1999). However, one of the limitations of the BoW model is that it disregards the semantics and context of words, which can lead to a loss of important information (Jurafsky and Martin, 2000). Despite these limitations, BoW remains a popular choice due to its simplicity and effectiveness in various applications.

#### 2.3.5 GloVe

Global Vectors for Word Representation (GloVe) is an unsupervised learning algorithm for obtaining vector representations for words (Pennington et al., 2014). Unlike traditional count-based methods such as the BoW or TF-IDF, GloVe leverages the global statistical information of a corpus. It constructs a co-occurrence matrix of words and captures the ratios of word co-occurrences to encode semantic relationships in a lower-dimensional space. This method allows GloVe to preserve linear substructures in the vector space, enabling analogical reasoning and capturing semantic similarities between words. GloVe has shown superior performance in various natural language processing tasks, including word analogy and word similarity benchmarks, and has become a popular choice for generating word embeddings that are used in downstream tasks such as text classification, machine translation, and sentiment analysis (Brochier et al., 2019).

The GloVe model effectively bridges the gap between count-based methods and predictive models like Word2Vec by combining the strengths of both approaches. While Word2Vec captures local context through sliding windows, GloVe integrates this with global statistical information, leading to more robust and meaningful word vectors (Levy and Goldberg, 2015). The ability of GloVe to capture both syntactic and semantic relationships between words is further enhanced by its ability to scale efficiently across large datasets, making it ideal for tasks that require high-quality word embeddings (Li et al., 2018). Additionally, GloVe's embeddings have been shown to perform well across different languages and domains, contributing to its widespread adoption in the NLP community for applications ranging from machine translation to question-answering systems (Bojanowski et al., 2017).

### 2.4 Prediction models

#### 2.4.1 Artificial Neural Network

Artificial Neural Networks (ANNs) are computational models inspired by the structure and functioning of biological neural networks. An ANN consists of layers of interconnected nodes, or neurons, that process and transmit information. These networks are typically organized in an input layer, one or more hidden layers, and an output layer (LeCun et al., 2015). Each neuron applies a nonlinear activation function to the weighted sum of its inputs, enabling the network to capture complex patterns in data. ANNs have been widely applied in various domains, including computer vision, natural language processing, and speech recognition, due to their ability to learn from data and generalize to unseen examples (Schmidhuber, 2015). One of the key advantages of ANNs is their ability to perform hierarchical feature extraction, where higher-level representations are built from lower-level features (Goodfellow et al., 2016). This makes ANNs particularly effective in tasks that involve high-dimensional and unstructured data.

#### 2.4.2 Random Forest

Random Forest (RF) is an ensemble learning method that operates by constructing a multitude of decision trees during training and outputting the mode of the classes (classification) or mean prediction (regression) of the individual trees (Breiman, 2001). The core idea behind Random Forest is to combine the predictions of multiple decision trees, each trained on a random subset of the data, to improve accuracy and control overfitting. This approach reduces variance by averaging the results, making RF highly robust against noisy data and overfitting, especially in high-dimensional spaces (Liaw and Wiener, 2002). Moreover, Random Forest provides an intrinsic measure of feature importance, which can be valuable in interpreting the model's decisions (Ho, 1998). Due to its versatility and performance, Random Forest has been widely adopted in various fields, including bioinformatics, finance, and remote sensing.

#### 2.4.3 Extreme Gradient Boosting

Extreme Gradient Boosting (XGBoost) is an advanced implementation of gradient boosting designed to enhance the performance and efficiency of machine learning models (Chen and Guestrin, 2016). XGBoost builds upon the principle of gradient boosting, where models are trained sequentially to correct the errors of previous models by optimizing a loss function. XGBoost introduces several innovations, including a regularization term to prevent overfitting, and efficient handling of sparse data and missing values (Chen et al., 2015). Furthermore, XGBoost is designed to be highly scalable, capable of running on distributed systems and handling large datasets with millions of examples (Zhang et al., 2017). Due to its ability to deliver high accuracy, speed, and scalability, XGBoost has become one of the most popular and widely used machine learning algorithms, particularly in competitive data science and applied machine learning.

#### 2.4.4 Support Vector Machine

Support Vector Machine (SVM) is a powerful supervised learning algorithm used primarily for classification tasks, but it can also be applied to regression problems (Cortes and Vapnik, 1995). SVM works by finding the optimal hyperplane that maximally separates data points of different classes in a high-dimensional space. The main objective is to maximize the margin between the nearest points of different classes, known as support vectors, to the hyperplane (Vapnik, 1998). This approach makes SVM highly effective in high-dimensional spaces and well-suited for complex datasets where the classes are not linearly separable. To handle such cases, SVM employs the kernel trick, which implicitly maps input features into higher-dimensional spaces, enabling the algorithm to find non-linear decision boundaries (Schölkopf and Smola, 2002). Due to its robustness and high accuracy, SVM has been widely used in various fields, including text classification, image recognition, and bioinformatics.

### 2.5 LIME

Local Interpretable Model-agnostic Explanations (LIME) is a popular XAI technique designed to interpret predictions made by complex, black box ML models (Ribeiro et al., 2016b). LIME has been applied to enhance the interpretability of models that predict mental disorders from social media data. By providing explanations for individual predictions, LIME helps in understanding which features (words, phrases, or patterns) in the text contribute most to the detection of conditions like depression or anxiety. This transparency is crucial for validating the model's decisions and ensuring that they align with clinical knowledge and intuition. LIME is also valuable in educational settings and research. It aids in demonstrating the internal workings of machine learning models to students and researchers. For example, in research focusing on detecting depression from X data, LIME can be used to show the significance of specific keywords or patterns, facilitating a better understanding of the model's behavior and improving its design and accuracy (Guo et al., 2023b).

By incorporating LIME into mental disorder detection models, researchers and practitioners can ensure that their models are not only accurate but also interpretable and trustworthy. This makes LIME a valuable tool in developing and deploying AI-based mental health diagnostics. The use of LIME enhances the transparency of machine learning models. In mental health applications, this transparency helps in gaining the trust of clinicians and patients, as they can see which features are influencing the model's predictions and assess whether these align with clinical expertise and evidence (Khoo et al., 2024; Akhtar et al., 2025).

## 3 Literature review

The detection of mental disorder through social media content is garnering significant attention from the research community (Santos et al., 2023; Amanat et al., 2022a; Chanda et al., 2022; Ji et al., 2022a; Haque et al., 2022; Wani et al., 2022; Rizwan et al., 2022; Ramírez-Cifuentes et al., 2021; Ghosh and Anwar, 2021; Mohammed et al., 2021). Table 1 provides a summary of related studies in the literature on mental disorder detection using machine learning and NLP techniques.

**Table 1:** Comparison table of some literature review.

**Study**	**Data source**	**Methods and accuracy**
Helmy et al. (2024)	English & Arabic Tweets	TF-IDF, BOW Lgbm 96.3%, RF 95.7% L-svm 95.9%, Rbf-svm 20%, LR 96.4%
Santos et al. (2023)	Twitter/X	LIWC 58%, 67%, 56%
Adarsh et al. (2023)	Reddit	SVM + KNN 98.05%, SVM 84.92%, DT 86.16%, RF 86.64%, XGBoost 88.48%, CNN 89.42%
Kabir et al. (2023)	Twitter/X	SVM 51%, 51%, 51%, 54% BiLSTM 62%, 56%, 79%
Amanat et al. (2022a)	Text Tweets	RNN 99%
Chanda et al. (2022)	Twitter/X	SVM 71%, KNN 62%, RF 54%, DT 52%
Ji et al. (2022a)	Twitter/X & Reddit	CNN 78%, LSTM 80%, BiLSTM 82%, RCNN 80%, SSA 81%, RN 83%
Haque et al. (2022)	Twitter/X	ML 93%, TN 94.0%, TN 92.5%, BiLSTM 93%
Saha et al. (2022)	Twitter/X	CNN 41%, RU44%, LSTM 45% Bi-GRU 41%, Bi-LSTM 41%
Wani et al. (2022)	Twitter/X, FB Youtube	CNN 98.15%, Word2Vec LSTM 92.19% CNN + LSTM 91.48%
de Souza et al. (2022)	Reddit	LSTM 65%, CNN 79%, Hybrid 72%
Tong et al. (2022)	TTDD, CLPsych 2015 LSVT, Statlog, Glass	86%, 85%, 87%, 86%, 87%, 87%
Santhosh Baboo and Amirthapriya (2022)	Twitter/X	RF 73%, LR 77%, SGB 72%
Zhang T. et al. (2022)	Tweets based	CNN 17%, RNN 36%, Transformer based methods 17%, hybrid-based methods 30%
Jain et al. (2022)	Reddit	NB 74.35%, SVM 77.12% LR 77.29%, RF 77.29%
Ramírez-Cifuentes et al. (2021)	Reddit	88%
Ghosh and Anwar (2021)	Twitter/X	Depression score 91%
Mohammed et al. (2021)	Bangala Data	DT 81.56%, RF 91.64%, AB 85.12% XGB 92.80%, GNB 91.06%, MLP 87.29%
Hemmatirad et al. (2020)	Twitter/X & Reddit	Twitter/X 95%, Reddit 73%
Tlachac and Rundensteiner (2020)	Twitter	CNN 86%, LSTM 90%, Naive Bayes 82% NN-BiLSTM with Attention model 97%
Fatima et al. (2020)	eRisk 2018	LR 76%, NB 67%, SVC 67%
Tadesse et al. (2019)	Reddit & Twitter/X	91%

More recently, Ibrahimov et al. (2025) emphasized model transparency alongside performance, introduced Depression X, a knowledge-infused residual attention model achieving a 7% F1 improvement while providing interpretable insights. Similarly, Qasim et al. (2025) utilized transformer-based architectures (e.g., BERT/RoBERTa) to assess depression severity directly from social media text. In another study, Friedman et al. (2024) proposed EAC-Net, an emotion-aware encoder leveraging contrastive learning and self-attention, demonstrating superior recall on depression and stress detection across multiple datasets. Expanding into multimodal input, Cha et al. (2024) presented MOGAM, which integrates video, text, and metadata via graph-attention mechanisms, achieving 0.87 accuracy on clinically labeled users. Additionally, Al Asad et al. (2024) developed a BERT+Bi-LSTM pipeline for both English and Arabic, highlighting the importance of explainability in achieving top F1 scores across languages.

In another work, Ibrahimov et al. (2024) highlighted the critical role of XAI frameworks in making mental health AI models transparent and trustworthy. Moving beyond surveys, Chen and Lin (2025) developed LLM-MTD, a large-language-model based multi-task system that simultaneously classifies depression and generates medically informed explanations, achieving state-of-the-art results on the RSDD benchmark. Empirical studies, such as those by Hoque et al. (2025), demonstrate the effective application of explainability tools like SHAP and LIME in real-world educational datasets. Their model attained over 91% accuracy in detecting depression in Bangladeshi university student posts, reinforcing the value of interpretability in high-risk settings.

Tadesse et al. (2019) proposed an approach to identify depression-related posts on Reddit using NLP and ML techniques. Their approach highlighted the significant improvement in detection accuracy by using a combination of linguistic features and classifiers, achieving up to 91% accuracy with a Multilayer Perceptron (MLP) classifier. Their work underscores the importance of feature selection and combination in enhancing the performance of depression detection systems. Furthermore, Guntuku et al. (2017) explored the detection of depression through social media data, highlighting significant advances in NLP and ML that facilitate large-scale mental health screening. Despite these technological advances, the generalizability of such studies to diverse populations and alignment with established clinical criteria remains uncertain. Furthermore, recent work highlighted the pressing need to address ethical, legal, and clinical considerations, particularly concerning data ownership, privacy protection, and the integration of these methods into existing healthcare systems.

Cho et al. (2019) proposed an approach to a comprehensive evaluation of the research that has been conducted on the use of ML algorithms for the diagnosis of depression, as well as recommendations for the practical uses of ML and predicted clinical remission following treatment with citalopram for twelve weeks. The dataset consisted of 1949 sad individuals who were participating in level 1 of the Sequenced Therapy Options to Relieve Depression study. In analyzing mental health using ML techniques, the primary focus is on providing a supervised learning environment for classification.

Another work by Helmy et al. (2024) explored the application of machine learning techniques to identify signs of depression in X data. It introduces manually labeled Arabic and automatically labeled English depression corpora, evaluates various pre-processing, feature extraction, and supervised classification techniques, and demonstrates the viability of machine learning for early depression detection despite recent trends favoring deep learning. This work underscores the importance of diverse, language-specific corpora and provides valuable insights into effective combinations of methodologies for predicting depression severity. The experiments demonstrated the significant impact of feature representation and resampling techniques on classifier performance, with Random Forest (Breiman, 2001) and RBF-SVM (Schölkopf and Smola, 2002; Cortes and Vapnik, 1995) models showing high effectiveness across different scenarios.

Santos et al. (2023) proposed the use of Mixture of Experts models combined with BERT-based approaches for predicting depression and anxiety from self-reports on social media in Portuguese was proposed. Their findings indicate that models outperform traditional feature engineering methods while also allowing for more interpretable models. The finding suggests potential improvements through modifications such as attention mechanisms, hierarchical mixtures, and multi-task learning. More recently, Amanat et al. (2022a) investigated the application of deep learning models for detecting signs of depression in textual data sourced from social media. The proposed framework leveraged LSTM networks and RNNs to analyse and classify text, achieving an impressive accuracy of 99% in early depression detection. The findings underscore the potential of advanced machine learning techniques to enable timely and precise identification of depressive tendencies, offering valuable support for mental health interventions and early assistance strategies.

Guo et al. (2023b) investigated mental health detection using text data from online forums, employing advanced machine learning techniques, including CNNs and LSTM networks. These models captured complex patterns and contextual nuances in textual data. To enhance the interpretability of these inherently black box models, the authors used the LIME technique. The LIME provided insights into the specific language patterns and features that influenced the model's predictions, enabling researchers to link certain textual expressions to mental health conditions. The interpretability increased the model's trustworthiness and supports its integration into clinical settings, where understanding decision rationale is critical for adoption and application.

Furthermore, Joyce et al. (2023) introduced the TIFU framework to enhance the trustworthiness of AI in psychiatry by focusing on transparency and interpretability. The author emphasized the importance of explainable AI, particularly through methods like LIME, to make complex models more understandable for healthcare professionals and patients, enhancing their reliability and acceptance in mental health applications. Abd Yusof et al. (2017) developed a computational model to identify potential causes of depression by analyzing user-generated content. This work identified prominent causes of depression and how they evolved, highlighting differences between individuals with varying levels of neuroticism. Another study, Sabaneh et al. (2023) integrated several advanced methodologies, including the use of ChatGPT-3 for translating Arabic text to English, QuickUMLS (Soldaini and Goharian, 2016) for extracting medical concepts from the translated text, and machine learning algorithms for classification. The researchers utilized a variety of classification algorithms, such as RF, SVM, and LR, with RF achieving the highest accuracy of 80.24%.

Saxena et al. (2022) explored the challenge of multi-class causal categorization of mental health issues on social media, focusing on the problem of incorrect predictions due to overlapping causal explanations. Their work identified inconsistencies in causal explanations as a key reason for varying accuracy by fine-tuning classifiers and applying LIME and Integrated Gradient methods (Sundararajan et al., 2017; Kokhlikyan et al., 2020; Ancona et al., 2018). The proposed approach was validated on the CAMS dataset, achieving category-wise average scores of 81.29% and 0.906 using cosine similarity and word mover's distance, respectively. Furthermore, Adarsh et al. (2023) utilized LIME to enhance the explainability of their classification model. The LIME was employed to identify and highlight specific words within social media posts that significantly contribute to the classification of posts as either containing suicidal ideations or not. The LIME was used in their approach to enhance the transparency and interpretability of the depression detection model, making it easier to understand and trust the model's decisions, particularly in identifying critical language markers of suicidal ideation.

## 4 Methods

**Figure 1:**
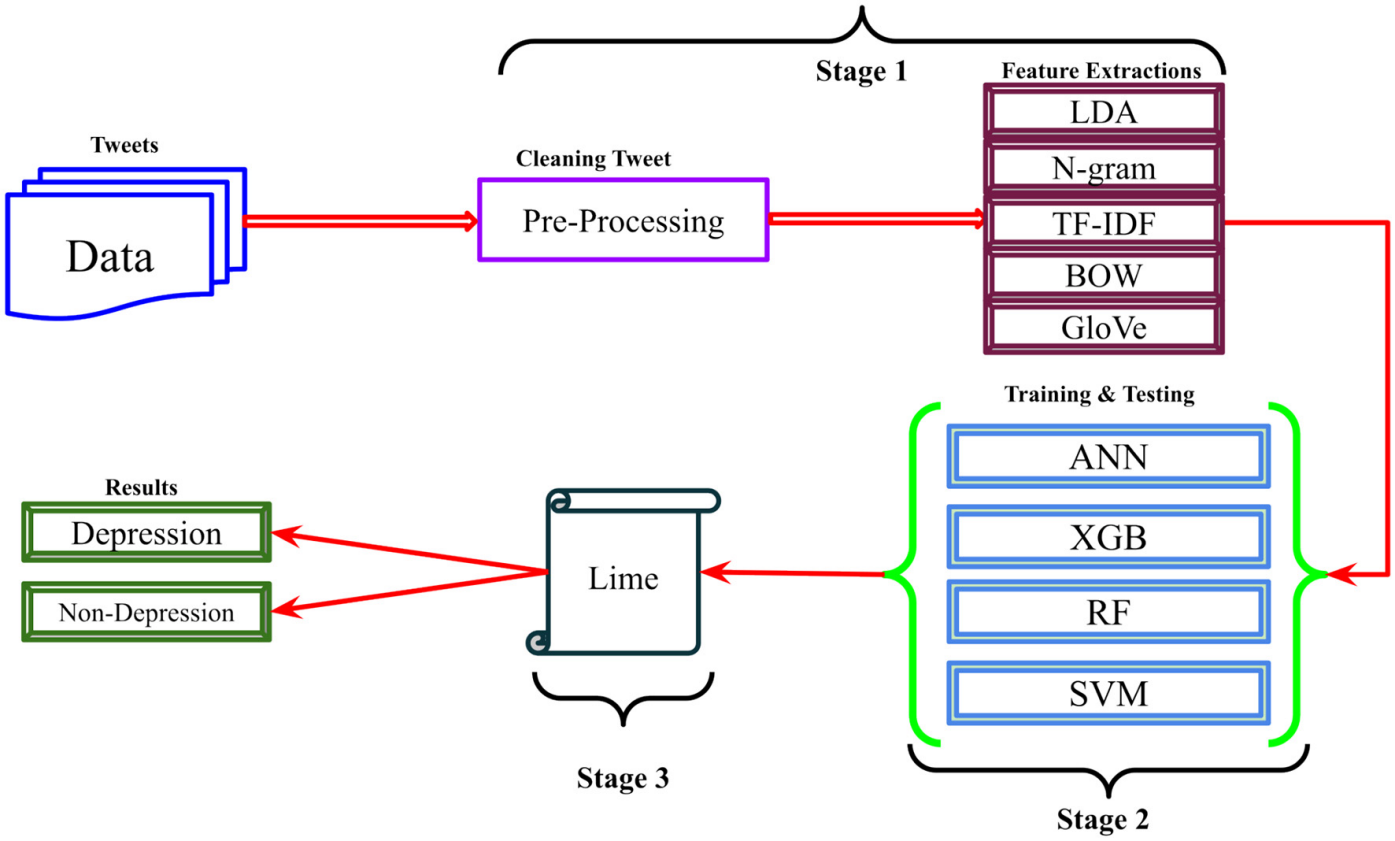
The proposed research methodology for depression detection using NLP and XAI techniques.

This research work presents a robust approach for detecting depression from X posts. The proposed approach consists of three steps: First, preprocessing techniques and feature extraction methods; second, machine learning classifiers; and third, interpretability analysis using LIME. The comprehensive methodology is depicted in Figure 1.

### 4.1 Data collection

The reliability and accuracy of any proposed system are intrinsically linked to the quality and representativeness of the data collected. As such, data serves as the cornerstone of the system's overall effectiveness and performance (Jain et al., 2020). Therefore, the collection and preparation of an appropriate dataset are essential to achieve the desired objectives. The experiments conducted in this work utilized publicly available datasets hosted on Kaggle^1^. In this work, we utilize a dataset derived from posts and comments on X. The analysis focuses on key factors, such as indications of mental illnesses like depression, as reflected in user posts and interactions. Representative instances of the data set are presented in Table 2 for illustrative purposes. Table 3 demonstrates the words for the posts in both categories, like “depression” and “non-depression” which are topically specific.

**Table 2:** Labeled instances from the X dataset used for classification.

**User**	**Tweet**	**Class**
Scotthamilton	Is upset that he can't update his Facebook by texting it... and might cry as a result School today also. Blah!	Non Depression
Mattycus	@Kenichan I dived many times for the ball. Managed to save 50% The rest go out of bounds	Non Depression
BaptisteTheFool	Meh... Almost Lover is the exception...this track gets me depressed every time.	Depression
erika_strange	@infidelsarecool ugh how depressing. i want to punch something.	Depression
ACTinglikeamama	@gigdiary I know - was a little depressed that we ate so much last night there were no leftovers today	Depression

**Table 3:** Words frequently used in depressive text.

**Depression**	**Non depression**
Alone, break, blame, depressed,	Go, days, aww, almost, holiday
Unhappy, worry, exam, rubbish	UK, son, YouTube, liked, Chicago
Danny, office, upset, past, reason	Happy, dreamy, love, Faith, Games
Needs, dead, hmmm, random, sd	Awsome, money, Movie, Frnds, hills
Waiting, hurt, blocked, cry, lost	Really, half, mad, episode, loved
Headache, summer, death, sucks	Lucky, cute, girls, town, visit
Miley Cyrus, job, Painfull, Massive	Needs, rest, excited, joy, happy
Upset, kick, dumb, Unsuccessful	Haha, listening, high, puppy, oooh
Disappointed, kill, Sadly, end	Went, ago, finished, drink, milk
STILL, feeling, busy, dark, migraine	DAMN, please, play, song, dance

Posts were included in the dataset if they met specific inclusion criteria: they had to be written in English, contain at least five words to ensure sufficient linguistic context for natural language processing (NLP), and include depression-related keywords such as “depressed,” “sad,” or “alone,” as identified in prior research as indicators of mental distress on social media (Chancellor and De Choudhury, 2020). Conversely, exclusion criteria were applied to remove posts that could compromise the reliability of the model. These excluded posts containing only emojis, links, or hashtags due to their lack of semantic and syntactic depth, advertisements or spam-like content that do not represent authentic user emotions and introduce noise, and explicitly sarcastic or humorous content, which may distort model predictions due to linguistic ambiguity.

For this work, a total of 1,600,000 tweets were collected, representing a diverse spectrum of user experiences related to critical factors such as depression and other mental health conditions. The data collection process involved filtering tweets using keywords indicative of mental health issues, including terms such as “depressed,” “anxiety,” and other relevant expressions.

### 4.2 Data pre-processing

**Figure 2:**
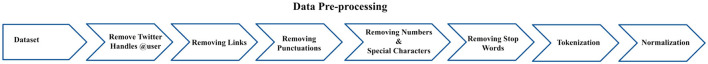
Data pre-processing.

Before feature selection and model training, NLP techniques were applied to pre-process the collected dataset. The initial step involved cleaning X posts from the data, resulting in a substantial dataset ready for feature extraction. The data pre-processing steps are illustrated in Figure 2.

The following pre-processing steps were implemented:

Tokenisation: the process of tokenization involves splitting the textual data into individual units, typically words or tokens, to facilitate further linguistic analysis. This step enables the transformation of unstructured text into a structured format suitable for feature extraction and modeling.

Noise removal: to enhance the quality of the dataset, noise removal techniques were applied. This included eliminating irrelevant elements such as URLs, punctuation marks, numerical values, and common stop words that do not contribute a significant semantic meaning. By refining the data set in this way, the subsequent analysis becomes more focused and meaningful.

Stemming: stemming techniques were employed to reduce words to their root or base forms, thereby minimizing variations of the same word (e.g., “running” and “ran” both reduced to “run”). This step helps to normalize inflected words and consolidate similar terms, leading to a more compact and informative feature space.

Normalization: normalization was carried out by converting all text into lowercase, ensuring uniformity across the dataset. This step prevents the algorithm from treating words with different cases (e.g., “Text” vs. “text”) as distinct entities, thereby improving the consistency and reliability of the text representation.

### 4.3 Feature extraction

After pre-processing, we employed several standard feature extraction techniques to capture the linguistic and semantic characteristics of user-generated posts. To reduce feature dimensionality while preserving document-level semantic structure, LDA was applied, modeling 70 latent topics. The TF-IDF vectors were generated to weight word importance across the corpus, facilitating the identification of salient terms. Both unigrams and bigrams were extracted using the Scikit-learn library, limiting to the top 3000 most frequent n-grams to improve contextual understanding in short texts. Additionally, the BoW representation was used as a baseline, relying on sparse word counts without considering syntactic relationships. Finally, pre-trained GloVe embeddings were incorporated to capture semantic similarity and contextual relationships between words in dense vector form. These diverse feature representations were used as inputs for training multiple machine learning models for classification.

The selection of feature extraction methods in this study was guided by two key considerations. First, we aimed to include a mix of traditional lexical representations (TF-IDF, N-gram, BOW), topic modeling approaches (LDA), and dense vector embeddings (GloVe) to capture both surface-level and semantic aspects of text. This diversity allows us to evaluate how LIME explanations differ when models are trained on features with fundamentally different representational properties. Second, we selected methods that are widely used in prior depression detection and sentiment analysis research, enabling meaningful comparison with existing literature and ensuring reproducibility.

While alternative embedding methods such as FastText, Word2Vec, or contextual embeddings like BERT are available, GloVe was chosen because it offers strong semantic representation with relatively low computational cost, making it suitable for large-scale experiments across multiple classifiers. Additionally, GloVe embeddings are static, which ensures that any interpretability differences observed using LIME are attributable to the model and feature-classifier interaction, rather than dynamic embedding variability. This controlled setting aligns with our goal of producing a consistent, explainability-focused benchmark rather than exhaustively comparing all possible embedding types.

GloVe was selected as the representative dense vector embedding method in our study for several reasons. First, GloVe captures global co-occurrence statistics, allowing it to encode semantic relationships between words effectively, an important factor for short-text, depression-related posts, where subtle semantic cues may indicate emotional state. Second, its extensive prior use in depression detection and sentiment analysis literature ensures comparability with existing work. Third, preliminary trials on our dataset indicated that GloVe produced slightly higher accuracy and more consistent performance across classifiers compared to FastText, whose subword-level advantages were less pronounced in our data due to the prevalence of short, informal tokens. By including GloVe alongside statistical (TF-IDF, N-gram, BOW) and probabilistic topic-modeling (LDA) methods, we aimed to evaluate LIME explainability across a diverse spectrum of feature representations.

In literature, TF-IDF is widely used to identify term importance in documents; it suffers from known drawbacks: high-dimensional, sparse vector representations, and an inability to capture semantic relationships such as synonymy or context, especially in short texts like tweets (Xu et al., 2013; Joshi et al., 2020). To address these issues, we limited the TF-IDF vocabulary to the top-N frequent terms (e.g., top 5,000) to reduce dimensionality and noise, following best practices in short-text feature selection. We also incorporated semantic embeddings such as GloVe to enrich the representations with contextual meaning beyond term frequency. Finally, we employed LIME to provide *post-hoc* interpretability, helping validate and visualize influential terms that drive classification decisions in our depression detection models.

### 4.4 Model training and validation

We divided the dataset into three distinct subsets: training, validation, and testing. The training set comprised 70% of the total data, amounting to 1,120,000 tweets, while the validation and testing sets included 15% each, consisting of 240,000 tweets for validation and 240,000 tweets for testing. This division allows us to effectively develop and fine-tune our models while ensuring an unbiased evaluation.

The class labels “depression” or “non-depression” were intuitively assigned based on the presence of specific depression-related keywords in the tweets, such as “depressed,” “sad,” “cry,” and “alone.” These labels were generated using a perception-based weak labeling approach, which is common in social media mental health detection studies. While this approach facilitates large-scale data collection, it may introduce noisy labels, which we mitigated through extensive pre-processing and validation using multiple classifiers. We acknowledge this limitation and highlight it in our Discussion section, suggesting the integration of expert-driven annotation in future work.

Table 4 outlines the key hyperparameters and settings used for each classifier. These values were chosen based on iterative testing on the validation set, informed by prior literature and practical experimentation.

**Table 4:** Classifier hyperparameters used for depression detection, selected through empirical tuning and standard practices.

**Model**	**Parameter**	**Value/setting**
ANN	Input layer	TF-IDF or GloVe embeddings
	Hidden layers	Two: 64 and 32 neurons
	Activation	ReLU (hidden), Softmax (output)
	Optimizer	Adam (learning rate = 0.001)
	Loss function	Categorical Cross-entropy
	Epochs/batch size	20/32 with early Stopping
SVM	Kernel	Linear
	Regularization (C)	1.0
	Decision function	One-vs.-rest
Random forest	Number of trees	100
	Max depth	None (expand until pure)
	Criterion	Gini Index
	Bootstrap	True
XGBoost	Number of estimators	100
	Max depth	6
	Learning rate	0.1
	Objective	Binary:logistic

To check how well our models worked, we used a common method called hold-out validation. We split the dataset into 80% for training and 20% for testing. The test data was not used during training or tuning, so we could see how well the model performs on new, unseen data. We also used five-fold cross-validation while training models like SVM, Random Forest, and ANN. This method helps us fine-tune model settings and reduce the chance of overfitting. To measure performance, we used standard metrics like accuracy, precision, recall, and F1-score. We also created confusion matrices and ROC curves to visualize how the models performed. These results were all based on the test data to make sure they were reliable. For the ANN, we used early stopping to avoid overfitting. This means the training stopped automatically when the model stopped improving on the validation data.

To classify tweets as indicative of depression or not, we selected and implemented several well-established machine learning models that have demonstrated strong performance in text classification tasks. We utilized the ANN, specifically a Multi-Layer Perceptron (MLP) architecture with two hidden layers of 4 and 16 neurons, respectively, as this provided a manageable level of complexity for evaluating various feature representations. The RF algorithm was selected for its robustness against noisy data and its ability to mitigate overfitting through the aggregation of multiple decision trees and random feature selection. We also employed XGBoost, chosen for its superior accuracy and regularization capabilities, which are achieved by sequentially optimizing weak learners to minimize classification error. Additionally, we incorporated the SVM because of its effectiveness in handling high-dimensional feature spaces, utilizing kernel methods to identify optimal separating hyperplanes. Each of these classifiers was trained using the same preprocessed and vectorized data, ensuring a fair and consistent comparison of their respective performance in detecting depression from tweets.

### 4.5 Performance evaluations

We assessed the performance of the model by applying well-known performance metrics, including accuracy and precision, and recall. The formulas of these evaluation metrics are shown below:


(7) $$\begin{array}{l} \text{Accuracy}=\frac{TP+TN}{TP+TN+FP+FN} \end{array}$$

(8) $$\begin{array}{l} \text{Precision}=\frac{TP}{TP+FP} \end{array}$$

(9) $$\begin{array}{l} \text{Recall}=\frac{TP}{TP+FN} \end{array}$$

(10) $$\begin{array}{l} F1=\frac{2\times \text{Precision}\times \text{Recall}}{\text{Precision}+\text{Recall}} \end{array}$$

## 5 Results

The robustnsess of the proposed approach lies in our preprocessing pipeline, which refers to its ability to effectively clean and normalize noisy, informal social media text, including irregular spellings, emoticons, repeated characters, and abbreviations which are common on platforms like X (Chancellor and De Choudhury, 2020; Steinkamp and Cook, 2021b). This pipeline improved the quality of feature extraction by reducing vocabulary sparsity and enhancing model generalizability. Our approach also ensured consistent performance across all tested classifiers (ANN, SVM, XGBoost, RF), demonstrating resilience against data variation, which is crucial when working with user-generated, unstructured data. Compared to existing methods (e.g., Ji et al., 2022a; Amanat et al., 2022a), our framework achieves higher or comparable accuracy using simpler architectures (e.g., GloVe+RF: 88%, SVM+TFIDF: 79%), while maintaining interpretability through LIME, which is rarely integrated in similar works (Ji et al., 2022b; Amanat et al., 2022b). This hybridization of multiple NLP features with black box models, accompanied by transparent explanations, offers a practical and explainable solution for early depression detection. Furthermore, our system does not require deep learning or transformer-based models, making it more computationally efficient and suitable for real-world deployment.

**Figure 3:**
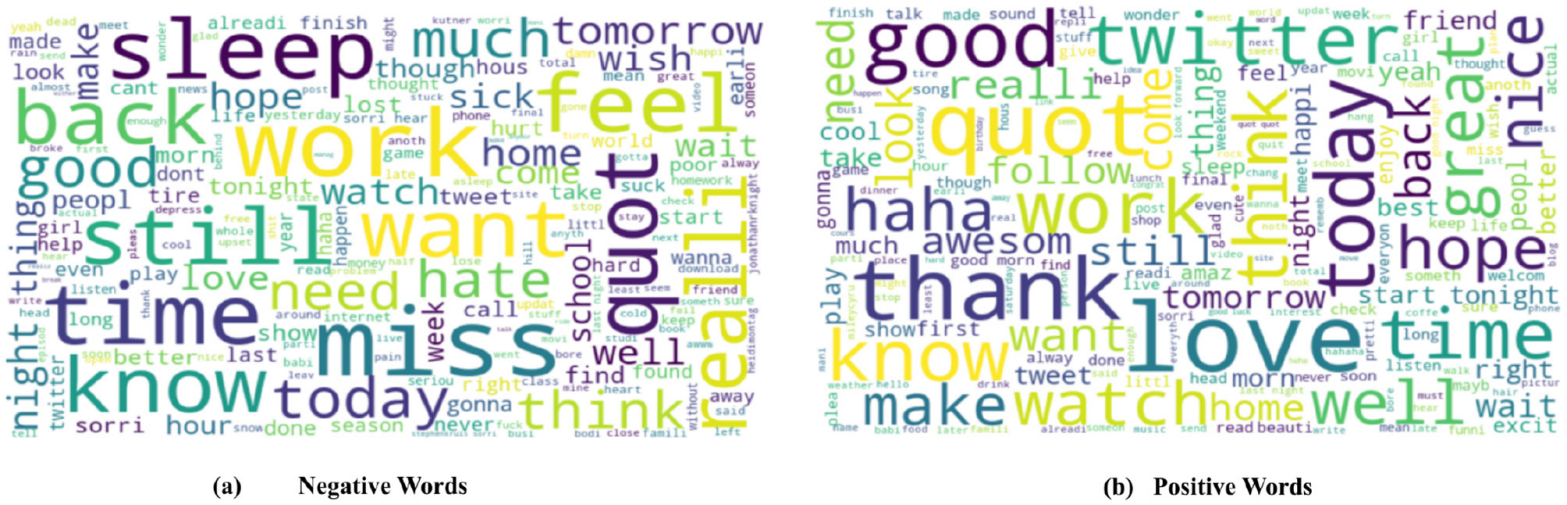
Word cloud for training data to visualize important. **(a)** Negative and **(b)** positive words.

Once the performance of the ML model has been evaluated, it becomes essential to analyse and interpret the findings to gain deeper insights into the model's performance. This involves discerning the crucial features influencing the model's predictions, comprehending the relationships between these features and the target variable, and identifying any pertinent patterns or trends within the dataset. This work employed a comprehensive set of experiments to detect depression from X posts using various combinations of feature extraction methods and ML classifiers. To visualize the most prominent terms that express emotions, a word cloud representation is utilized. Figure 3a demonstrates the depressive user's feelings, experiences, and stories. This method summarizes the ideas and phrases most commonly linked to mental disorders in the analysis of social media discussions. Figure 3b illustrates the word cloud representing the positive core words of the dataset. Word clouds are visual representations that draw attention to the most prevalent terms in a collection of text. In addition, the prominence of a word in the cloud reflects its frequency of use in the corresponding tweets. This method summarizes the ideas and phrases most commonly linked to mental disorders in the analysis of social media discussions. On the contrary, Table 5 presents the words correlated with the specific topics generated from the posts. These topics comprise a lexicon of words commonly used among accounts associated with depression.

**Table 5:** Topics extracted with LDA.

**Sr**	**Topics**	**Words**
1	Daily activities	Haha, play, friend, year, stop haha play friend year stop yeah tell think today chang want left know word
2	Planning:	Hous, damn, love, plan, like hous damn love plan like trip time dear usual watch lost today tomorrow cook list want
3	SocialMedia usage	Gonna, enjoy, thing, fun, welcome gonna enjoy thing fun welcom weather X love come cool sure
4	Home life	Home, week, away, head, update home week away head updat night guess today stuck bought outside
5	Depression	Good, feel, morn, better, hope good feel morn better hope morning right like hate night realli make today coffe bed think sleep class
6	Affection nostalgia	Readi, pretti, miss, yay, love readi pretti miss yay love song tomorrow goodnight saturday amp sleep hang alreadi night
7	Planning anticipation	Day, ll, someth, think, later day ll someth think later start make beauti tomorrow happen days amp let money
8	Work productivity	Work, glad, time, snow, home work glad time snow home hard today earli okay tonight easter night
9	Celebrations greetings	Happi, birthday, wonder, peopl, mani happi birthday wonder peopl mani love best sorri repli realli sooo
10	School life	Today, school, life, break, lunch today school life break lunch hour watch hear spring funni till wanna room
11	Quotations humor	Quot, listen, cold, like, hahaha quot listen cold like hahaha music babi love problem hey th great haha brother smile song
12	Love relationships	Love, tweet, nice, tire, amp love tweet nice tire amp awesome twitter summer train join kinda ddlovato
13	Happy	Awesom, Watch, like, send, wear awesom watch like send wear place love asot400 way help house man make amp fail
14	Technology	Know, need, want, twitter, hello know need want twitter hello dont think phone like love realli mileycyru fm cute
15	Reading learning	Read, alway, book, final, food read alway book final food time gone dinner believ think iphon famili tonight pick
16	Weekend activities	Good, weekend, girl, sick, luck good weekend girl sick luck night rain dream wish fuck shower tuesday great time today
17	Online engagement	Http, com, thank, follow, twitpic http com thank follow twitpic www tinyurl thanks check twitter link
18	Future plans	Time, watch, soon, movi, long time watch soon movi long suck come today love want realli heard movie anoth real
19	Sleep relaxation	Great, sleep, time, post, hope great sleep time post hope bore bit late everyth ly free breakfast http day
20	Visuals photos	Look, like, wait, forward, picture look like wait forward pictur sound realli think ll gt welcome twitter awww

The results of research experiments are summarized in Table 6, which shows the accuracy and prediction probabilities for each combination of feature extraction method and classifier. Additionally, Table 7 provides a comparative analysis of several ML classifiers, namely ANN, XGBoost, RF, and SVM, using different feature extraction techniques. These features include LDA, TF-IDF, N-gram, BOW, GloVe, and various combinations of these techniques. The classifiers are evaluated based on their performance metrics: Precision, Recall, and F1-score.

**Table 6:** Results of classification performance ML models and feature selection methods based on accuracy.

**Sr**	**Features**	**ANN**	**XGBoost**	**RF**	**SVM**
1	LDA	72	68	72	72
2	TF-IDF	72	77	78	79
3	N-gram	73	78	76	77
4	BOW	73	78	75	78
5	GloVe	72	86	88	85
6	LDA+TFIDF+Ngram	78	77	72	72
7	LDA+BOW+TFIDF	76	77	77	78

**Table 7:** Comparison of ML models and feature selection methods based on additional metrics such as precision, recall, and F-score.

		**ANN**			**XGBoost**			**RF**			**SVM**		
**Sr**	**Features**	**Prec**.	**Rec**.	**F1**	**Prec**.	**Rec**.	**F1**	**Prec**.	**Rec**.	**F1**	**Prec**.	**Rec**.	**F1**
1	LDA	73	94	82	74	87	80	74	94	81	72	98	83
2	TF-IDF	82	80	81	80	90	85	80	92	86	81	92	86
3	N-gram	83	79	81	81	91	86	81	86	84	81	94	86
4	BOW	83	81	85	81	91	85	82	84	83	79	94	86
5	GloVe	72	99	83	86	88	87	97	92	94	83	94	90
6	LDA+TFIDF+Ngram	80	91	85	82	87	84	79	92	84	79	92	84
7	LDA+BOW+TFIDF	82	85	84	78	94	86	77	95	85	79	94	86

It should be noted from Table 6 that the GloVe feature extraction technique combined with RF achieved the highest accuracy of 88%, demonstrating its ability to capture rich semantic information effectively. SVM also performed well with GloVe, achieving an accuracy of 85%. TF-IDF and N-gram modeling showed competitive performance, with XGB achieving an accuracy of 77% and 78%, respectively, demonstrating their effectiveness in capturing text features. BOW proved to be a reliable feature extraction method, with XGB and ANN yielding accuracies of 78% and 73%, respectively. The combination of LDA with TF-IDF and N-gram yielded an accuracy of 78% for ANN, showcasing the potential of combining multiple feature extraction techniques. Our experiments demonstrate the varied strengths of feature extraction methods and classifiers, with GloVe providing the most insightful semantic information. At the same time, N-gram and BOW maintained a consistent balance of performance across models.

**Figure 4:**
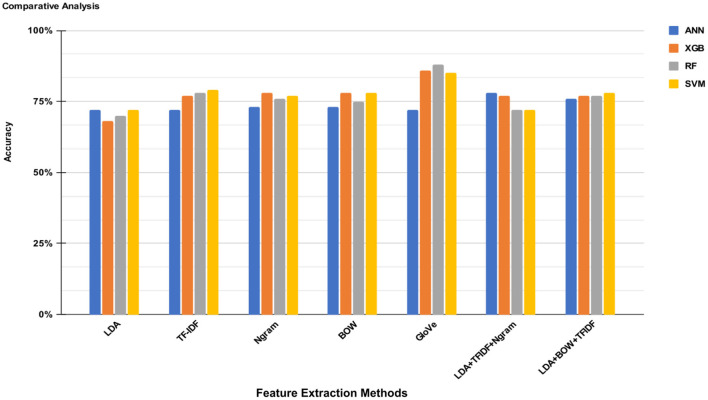
Comparison of ML models and feature selection methods based on accuracy.

Figure 4 presents a comparative analysis of four ML classifiers ANNs, XGB, RF, and SVM across various feature extraction methods: LDA, TF-IDF, N-gram, BOW, GloVe, LDA+TF-IDF+Ngram, and LDA+BOW+TFIDF. The performance of each combination is evaluated and depicted in terms of accuracy percentages. The highest performance is achieved by the GloVe feature extraction method, followed closely by the SVM algorithm at 86%. Overall, the GloVe method consistently outperforms other feature extraction techniques, indicating its effectiveness in capturing word semantics and improving classification accuracy. Other notable performances include the LDA+TF-IDF+Ngram method, which demonstrates balanced accuracy across different classifiers, particularly with SVM classifiers. This comprehensive comparison underscores the importance of selecting suitable feature extraction methods to boost the predictive power of machine learning models in text classification tasks.

### 5.1 Overview of evaluation

The experiments were performed on the same dataset for all feature extraction methods and classifiers to ensure consistent comparison. We evaluated seven feature extraction methods (LDA, TF-IDF, N-gram, BOW, GloVe, LDA+TFIDF+N-gram, and LDA+BOW+TFIDF) and four classifiers (ANN, XGBoost, RF, SVM). To improve analytical clarity, results are presented in two separate views: (1) ranking of feature extraction methods averaged across all classifiers, and (2) ranking of classifiers averaged across all feature extraction methods. Detailed per-configuration precision, recall and F1-score values are provided in the Tables 8, 9.

**Table 8:** Average accuracy of feature extraction methods across ML classifiers.

**Feature extraction**	**Accuracy (%)**	**Rank**
GloVe	82.75	1
LDA + BOW + TF-IDF	77.00	2
TF-IDF	76.50	3
N-gram	76.00	4
BOW	76.00	5
LDA + TF-IDF + N-gram	74.75	6
LDA	71.00	7

**Table 9:** Average accuracy of ML classifiers across feature extraction methods.

**Classifier**	**Avg accuracy (%)**	**Rank**
XGBoost	77.29	1
SVM	77.29	2
Random Forest (RF)	76.86	3
ANN	73.71	4

### 5.2 Feature extraction method rankings

Table 8 reports the average accuracy of each feature extraction method calculated over all four classifiers. This ranking shows which feature representations perform best on average in our study.

Interpretation: GloVe embeddings provide the highest average accuracy (82.75%) across classifiers, indicating that dense semantic representations capture context useful for depressive-linguistic signals in our dataset. Traditional lexical representations (TF-IDF, N-gram, BOW) remain competitive and may be preferable in resource-constrained settings.

### 5.3 Classifier rankings

Table 9 shows the average accuracy of each ML classifier across all feature extraction methods. This ranking isolates classifier performance independent of any single feature choice.

## 6 LIME analysis

Despite their excellent performance, several ML models are often characterized as black boxes that produce outputs without offering explicit insights into the underlying reasoning behind their decisions. Understanding and interpreting the decision-making processes of such models is critical, particularly in applications where trust, transparency, and accountability are paramount. Consequently, it is imperative to examine the outputs of these models thoroughly and, more importantly, to develop methodologies that enable the generation of interpretable explanations for their decisions (Søgaard, 2021; Gohel et al., 2021). Providing explanations for a model's output enhances our ability to evaluate its predictions critically, thereby fostering greater confidence in determining whether to trust or question its outcomes.

To investigate the model's explainability, we employed LIME, a popular XAI technique that facilitates the interpretation of outputs without requiring direct inspection of the model's internal structure. LIME achieves this by perturbing the local features surrounding a specific target prediction and analyzing the corresponding changes in the model's output. In our experiments, the words surrounding a target entity were modified systematically, and the effects on the model's predictions were subsequently assessed to gain insights into the decision-making process.

**Figure 5:**
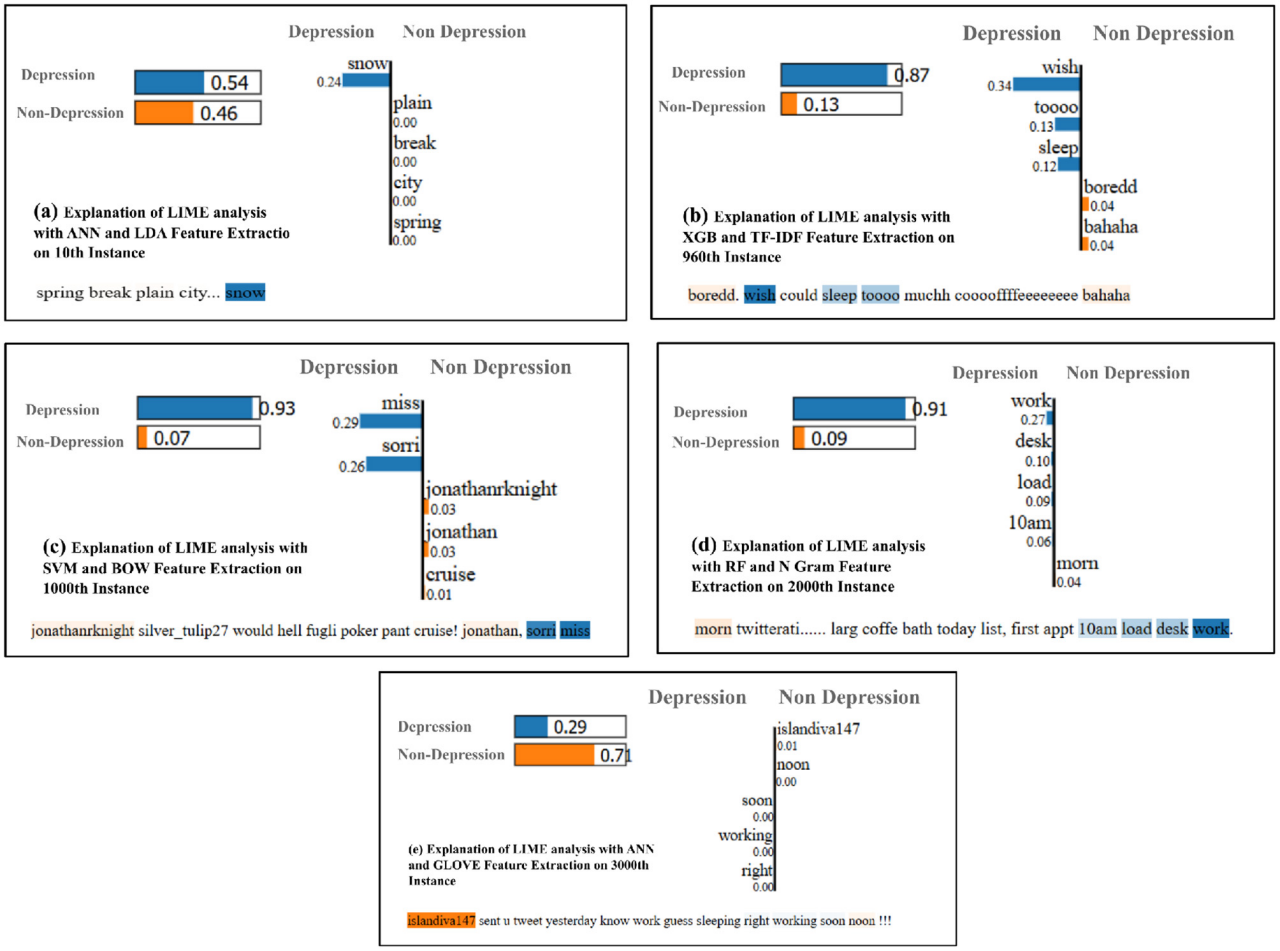
Example of explainable AI visualizations using LIME. **(A)** ANN and LDA feature extraction on 10th instance. **(B)** XGB and TF-IDF feature extraction on 960th instance. **(C)** SVM and BOW feature extraction on 1000th instance **(D)** RF and N gram feature extraction on 2000th instance. **(E)** ANN and GLOVE feature extraction on 3000th instance.

Each subplot in Figure 5 illustrates the LIME analysis visualizations, providing an interpretable explanation of the predictions made by different classifiers for specific instances. Each subplot highlights the contribution of individual words (features) toward the prediction of either “Depression” or “Non-Depression” labels. The importance of the words is represented as bars, where positive contributions toward “Depression” are shown in blue, and contributions toward “Non-Depression” are shown in orange.

Figure 5a demonstrates LIME analysis for ANN with LDA features extraction on the 10th instance. It can be noted that the word “snow” contributed significantly to the “Depression” label, with a probability score of 0.54, compared to 0.46 for “Non-Depression.” This shows how specific context-sensitive words can influence predictions. Similarly, Figure 5b demonstrates LIME analysis for XGB and TF-IDF on the 960th instance, the words “wish,” “sleep,” and “tooo” strongly contributed to the “Depression” label, achieving a high probability score of 0.87 for “Depression”. This highlights the model's ability to capture sentiment-relevant features using the TF-IDF approach.

In addition, Figure 5c demonstrates a dominant contribution of the words “miss” and “sorry” toward the “Depression” label, achieving a probability score of 0.93. This emphasizes the BOW model's capability to detect sentiment-related words. Similarly, Figure 5d visualizes that, RF with N-Gram on the 2000th instance, the words “work,” “desk,” and “10am” contribute strongly toward the “Depression” label, with a probability score of 0.91. This demonstrates how the N-Gram technique effectively captures contextual word co-occurrences. On the other hand, Figure 5e indicates that ANN with GloVe embeddings on the 3000th instance, provided a contrasting prediction, with the word “islandiva147” contributing more toward “Non-Depression” than “Depression.” This suggests that GloVe embeddings capture semantic nuances but may misinterpret words out of context.

Overall, these visualizations illustrate the interpretability of the classifiers and their reliance on feature-specific contributions. While ANN and SVM demonstrated strong performance on LDA and BOW features, respectively, XGB and RF highlighted the importance of TF-IDF and N-Gram features. GloVe embeddings, while semantically rich, occasionally misinterpret specific instances, underscoring the importance of feature selection.

## 7 Discussion

Our study examines various feature sets and classifiers, achieving notable accuracies, particularly when combining GloVe with classifiers such as RF and SVM. GloVe+RF, achieved 88% accuracy obtained by Ji et al. (2022a). In comparison, Amanat et al. (2022a) reported accuracies up to 96.4% using a combination of TF-IDF, BOW, SVM, and RF. While our results for GloVe+RF (88%) and GloVe+SVM (85%) are slightly lower, they are still competitive given the different data sources and methodologies used. which is within the range reported by comparable studies on depression detection using traditional machine learning approaches. Variations in reported accuracy across the literature are largely attributable to differences in datasets, preprocessing steps, and feature-classifier combinations, making direct score comparisons less meaningful. Instead, the emphasis here is on showing that competitive performance can be achieved alongside enhanced interpretability.

Among feature extraction methods, GloVe embeddings achieved the highest average accuracy across classifiers (82.75%), followed by LDA+BOW+TF-IDF (77.00%) and TF-IDF (76.50%). This indicates that semantic embeddings like GloVe capture richer contextual information than purely lexical representations. These results are consistent with prior work—for instance, Tong et al. (2022) reported 86% accuracy using GloVe-based features in a multi-classifier ensemble, whereas our GloVe+RF model reached 88% on this dataset. Although Amanat et al. (2022a) achieved higher performance (96.4%) with TF-IDF+BOW+SVM/RF, differences in datasets and preprocessing make direct comparisons indicative rather than conclusive. Notably, N-gram and BOW features, while ranking lower in average performance (76.00%), still matched or exceeded the accuracy of some deep learning models in the literature, such as the CNN (78%) and LSTM (80%) reported by de Souza et al. (2022), demonstrating that simpler representations can be competitive for certain datasets.

Among ML classifiers, XGBoost and SVM obtained the highest average accuracy across feature sets (77.29%), closely followed by Random Forest (76.86%), with ANN ranking lowest (73.71%). This suggests that tree-based ensembles and margin-based classifiers are generally better suited to the depression detection task when trained on short, noisy social media text. These trends align with the findings of Wani et al. (2022), where SVM achieved 71% and KNN 62% using N-gram features, and with Ji et al. (2022a), who reported competitive performance with SVM on short-text datasets. In our experiments, ANN performed best with the combined LDA+TF-IDF+N-gram feature set (78%), which slightly exceeds the BiLSTM performance (79%) reported by Haque et al. (2022), showing that under certain feature configurations, neural networks can still achieve strong results.

A key finding is that performance depends on the interaction between the feature set and the classifier. For example, while GloVe consistently ranks highest among features, its combination with RF (88%) and SVM (85%) outperformed its pairing with ANN (72%). Similarly, the LDA+TF-IDF+N-gram feature set worked particularly well with ANN (78%), but less so with RF (72%). These variations underscore the importance of evaluating both dimensions independently before selecting an optimal configuration.

Another distinctive aspect of this study is the systematic application of LIME across all feature-classifier combinations, rather than to a single model. LIME provided interpretable, instance-level explanations, identifying key linguistic cues such as first-person pronouns, negative emotion words, and self-referential phrases that heavily influenced depressive content predictions. This transparency is essential for building trust with mental health professionals and differentiates our work from most prior studies, where explainability is rarely addressed at this scale.

It should be noted that the accuracy of depression detection models heavily relies on the authenticity and consistency of users' social media content. Factors such as bias for social desirability, stigma, and personal tendencies can influence the way users express themselves online, potentially leading to inaccuracies in detection (Shah et al., 2025). In general, detecting depression from social media posts inherently depends on the assumption that users share relevant depressive symptoms or emotional cues in their online interactions. However, not all individuals with depression disclose their condition or express depressive symptoms publicly on social media, which presents a notable limitation of computational models relying solely on social media text. This limitation leads to potential false negatives, where affected individuals who do not manifest depressive behavior online may be missed by such systems.

Similarly, another recent study (Aldkheel and Zhou, 2024) highlighted that social media detection methods that focus on observable linguistic, visual, and behavioral signals indicative of depression cannot account for users who do not publicly share or mask their symptoms due to privacy concerns, social stigma, or personal choice. Moreover, reliance on textual content alone restricts detection to expressed emotions and behaviors, which may not comprehensively represent every user's mental health status.

The necessity for combining social media analysis with clinical validation and offline data is emphasized to address these limitations and improve detection reliability. Verification of ground truth through clinical evaluations or integration of medical records along with social media data can help overcome false negatives caused by the absence of explicit online depressive expression (Aldkheel and Zhou, 2024). Thus, while social media-based depression detection offers valuable early screening potential, it cannot substitute for comprehensive clinical diagnosis and does not capture all cases, especially among users who do not disclose symptoms online.

GloVe word embeddings worked better with models like Random Forest and ANN because they capture the meaning and relationships between words. Unlike methods like TF-IDF or Bag of Words that just count word frequency, GloVe places similar words (like “sad” and “unhappy”) close together in a way that shows their meaning. This helps the models better tell the difference between depressive and non-depressive posts, especially since social media posts are usually short. Traditional models like SVM and Random Forest did well with TF-IDF because they can handle large sets of sparse features. However, the ANN model didn't perform as well with TF-IDF or BoW since those features don't carry the deeper meaning that neural networks are designed to learn from. When we used LIME to explain the model's decisions, we found that GloVe helped the models focus on important words like “lonely,” “worthless,” and “help”— strong signs of depression. In contrast, TF-IDF often picked up common but less meaningful words, which made the learning less effective.

We used social media data from platform X (formerly Twitter) because it is public, real-time, and short in format making it ideal for spotting signs of mental health issues. Compared to sites like Reddit or medical records, Twitter has more variety in language, which helps our model work better in real-world situations (De Choudhury et al., 2013).

To get useful features from the text, we used both basic methods (like TF-IDF, LDA, N-gram, and Bag of Words) and word embeddings (like GloVe). While advanced models like BERT understand context better, they are slower and harder to explain. GloVe gave us good results without needing too much computing power, which is important if we want to use this in real-time systems (Pennington et al., 2014).

We picked simple models like SVM and Random Forest because they work well with smaller datasets, are faster to train, and are easier to understand—especially when used with tools like LIME. These models also don't need powerful hardware and can be used in apps or cloud platforms. We used LIME to explain how our models make decisions. It works with many kinds of models and helps make the results clearer for doctors and other users. This is important for mental health tools, where we need to be careful and ethical in how results are used (Ribeiro et al., 2016b).

Overall, our findings demonstrate the effectiveness of combining traditional and advanced NLP techniques with robust classifiers to achieve competitive performance in text classification tasks. Our results indicate that, while advanced deep learning models are powerful, conventional methods and hybrid approaches can also achieve competitive accuracy, offering more interpretable and computationally efficient alternatives.

To address the identified research gaps in the literature, our study proposes an interpretable and comparative framework for depression detection using Twitter data, comprising four key components. First, multiple NLP feature representations are employed, including TF-IDF, BoW, LDA, N-grams, and GloVe embeddings, to effectively capture both statistical and semantic characteristics of textual content. Second, a range of ML classifiers, including SVM, RF, ANN, and XGBoost, were evaluated under identical experimental conditions to assess their predictive performance. Third, explainability was integrated into the framework using the LIME method, which highlights the contribution of individual features to classification outcomes, thereby enhancing transparency and trust in the model. Finally, a comparative evaluation is performed in which all models and feature extraction techniques are applied to the same dataset and evaluated using consistent metrics, accuracy, precision, recall, and F1 score, to ensure fairness, reproducibility, and applicability in the real world.

## 8 Conclusion

Mental illness is a prevalent social issue driven by socioeconomic, clinical, and individual risk factors, and the rise of social media has allowed the analysis of user-generated content for early detection of depression. In this work, we evaluated the effectiveness of various feature extraction methods and machine learning classifiers in detecting depression from X posts. Our findings demonstrate that social media data can be effectively utilized for mental health monitoring, with methods such as N-gram, BOW, and TF-IDF providing significant insights. In particular, the combination of TF-IDF with XGB achieved the highest precision of 87%, while the GloVe embeddings with RF reached an accuracy of 88% with lower interpretability. The use of LIME highlighted the importance of balancing accuracy and interpretability in model outcomes. Future research should focus on incorporating advanced deep learning models such as Transformers and BERT, developing real-time detection systems, integrating multimodal data, expanding analyses to additional social media platforms, and addressing ethical and privacy concerns in mental health monitoring.

## Data Availability

The original contributions presented in the study are included in the article/supplementary material, further inquiries can be directed to the corresponding author.

## Footnotes

^1^ https://www.kaggle.com/datasets/adedamolaajewole/training1600000processednoemoticon
